# Transcranial alternating current stimulation combined with sound stimulation improves cognitive function in patients with Alzheimer’s disease: Study protocol for a randomized controlled trial

**DOI:** 10.3389/fnagi.2022.1068175

**Published:** 2023-01-09

**Authors:** Yang Liu, Shuzhen Liu, Can Tang, Keke Tang, Di Liu, Meilian Chen, Zhiqi Mao, Xuewei Xia

**Affiliations:** ^1^Department of Neurosurgery, Affiliated Hospital of Guilin Medical University, Guilin, China; ^2^Chengde Medical University, Chengde, China; ^3^Guangzhou Kangzhi Digital Technology Co., Ltd., Guangzhou, China; ^4^Department of Neurosurgery, Chinese PLA General Hospital, Beijing, China

**Keywords:** Alzheimer’s disease, cognition, transcranial alternating current stimulation, gamma rhythm, sound, clinical trial

## Abstract

**Background:**

The number of patients with Alzheimer’s disease (AD) worldwide is increasing yearly, but the existing treatment methods have poor efficacy. Transcranial alternating current stimulation (tACS) is a new treatment for AD, but the offline effect of tACS is insufficient. To prolong the offline effect, we designed to combine tACS with sound stimulation to maintain the long-term post-effect.

**Materials and methods:**

To explore the safety and effectiveness of tACS combined with sound stimulation and its impact on the cognition of AD patients. This trial will recruit 87 patients with mild to moderate AD. All patients were randomly divided into three groups. The change in Alzheimer’s Disease Assessment Scale-Cognitive (ADAS-Cog) scores from the day before treatment to the end of treatment and 3 months after treatment was used as the main evaluation index. We will also explore the changes in the brain structural network, functional network, and metabolic network of AD patients in each group after treatment.

**Discussion:**

We hope to conclude that tACS combined with sound stimulation is safe and tolerable in 87 patients with mild to moderate AD under three standardized treatment regimens. Compared with tACS alone or sound alone, the combination group had a significant long-term effect on cognitive improvement. To screen out a better treatment plan for AD patients. tACS combined with sound stimulation is a previously unexplored, non-invasive joint intervention to improve patients’ cognitive status. This study may also identify the potential mechanism of tACS combined with sound stimulation in treating mild to moderate AD patients.

**Clinical Trial Registration:**

Clinicaltrials.gov, NCT05251649. Registered on February 22, 2022.

## Introduction

The International Alzheimer’s Association reports that there will be an estimated 55 million people with dementia worldwide in 2021, and patients with Alzheimer’s disease (AD) are expected to increase to 78 million in 10 years (Chen et al., 2022). Thus, AD has become one of the major global health problems. The characteristic manifestations of AD are amyloid plaques composed of extracellular amyloid-β (Aβ) peptides and neurogenic fiber tangles composed of intracellular hyperphosphorylated tau proteins (Ferri et al., 2005; Jack et al., 2018). Although some therapies have been reported to eliminate the accumulation of Aβ plaques in the brain, improve symptoms and delay cognitive decline, they do not stop its progression (Tomita, 2017; Tolar et al., 2020; Rischel et al., 2022). Briefly summarizing the advances in AD treatment over the past decades, it is safe to say that the development of AD therapies has been extremely challenging.

AD is clinically defined as progressive cognitive decline and memory loss (Jack et al., 2018; Rischel et al., 2022). Cognitive decline is a primary concern in treating AD patients because of its impact on all aspects of their lives. A Cognitive Networks (cognit) consists, in and of, a net of cortical nerve cells and the fibers and synapses that connect them. The anatomical profile of a cognit is scattered and highly irregular, as its edges merge with weak or unstable connections to other related cognits. Depending on the complexity of its synapses and fibers, cognitive bodies vary widely in size and cortical coverage (Fuster, 2006, 2022; McNaughton and Vann, 2022). Cortical inputs are essential for maintaining working memory and delaying task performance, and conversely, damage to the lateral prefrontal cortex leads to working memory deficits and disruption of posterior association cortical cell activity (Hummos et al., 2022; Li et al., 2022; Zhang et al., 2022). These two phenomena manifest impaired “cognitive control” in the frontal lobe. Therefore, our present trial was designed to improve cognition in AD patients, starting from the prefrontal lobe.

Transcranial electrical stimulation (tES), performed through painless and non-invasive neuromodulation methods, has gained increasing interest as a potential non-pharmacological intervention for patients with Mild Cognitive Impairment (MCI) or AD (Mosilhy et al., 2022). tES includes transcranial direct current stimulation (tDCS) and transcranial alternating current stimulation (tACS). tDCS has a constant electric field and microcurrent across the scalp, which depolarizes [anodic tDCS) or hyperpolarizes (cathodic tDCS) the resting membrane potential of neurons tDCS], thus altering brain function (Marceglia et al., 2016; Duan et al., 2022). tACS is a newer form of non-invasive brain stimulation (NIBS) than tDCS and has the potential to be a promising alternative to tDCS (Senkowski et al., 2022). Because a growing body of literature has recently reported, the use of tACS can have an enhanced effect on different cognitive domains in healthy populations (Hoy et al., 2015; Santarnecchi et al., 2016; Gonzalez-Perez et al., 2019; Meier et al., 2019). tACS differs from tDCS in that it delivers alternating current between electrodes at specified frequencies in a bidirectional manner, whereas tDCS delivers unidirectional current (Reato et al., 2013; Fertonani and Miniussi, 2017). It was found that tACS at specific frequencies may lead to entrainment concussion at the target stimulation frequency (Kasten and Herrmann, 2017). Enhancing brain gamma-band wave activity can more directly enhance cognitive function or modulate the neuropathology of cognitive dysfunction (Kim et al., 2021; Lin et al., 2021; Yu et al., 2022).

The use of 40 Hz, a stimulation frequency, for the treatment of AD is a relatively hot topic. And most of the existing clinical studies using tACS to treat AD and dementia patients choose 40 Hz as the stimulation frequency. Gamma rhythms (35 Hz-48 Hz) are ubiquitous in the human brain and play a crucial role in memory function (Nyhus and Curran, 2010; Fries, 2015; Pina et al., 2018), and the involvement of gamma activity in various cognitive functions is a well-documented finding (Herrmann et al., 2004; Kaiser and Lutzenberger, 2005; Colgin and Moser, 2010; Carr et al., 2012). Gamma entrainment therapy can reduce the loss of functional brain connectivity and brain atrophy in AD patients, thereby improving cognitive function and improve several pathological markers (Traikapi and Konstantinou, 2021). Gamma band waves of brain electrical activity, especially 40 Hz oscillations, are closely associated with higher cognitive functions. Oscillations in this band activate microglia and clear amyloid-β deposits, and this effect is only present at 40 Hz, not at other frequencies (Iaccarino et al., 2016; Dhaynaut et al., 2022; Liu C. et al., 2022; Shen et al., 2022). Features of neurodegenerative diseases include abnormal protein aggregation, impaired protein degradation, failure of axonal transport, mitochondrial dysfunction, reduced energy metabolism, oxidative damage, and cell death (Mattson et al., 1999; Ardanaz et al., 2022). 40 Hz-tACS can address the AD pathophysiological cascade by modulating interneuronal activity that leads to global network dysfunction and activating microglia waste removal (Matsumoto and Ugawa, 2016). However, multiple frequencies of tACS (e.g., Delta, Theta, Alpha, Beta, and Gamma) have been reported to improve cognition in healthy people (Klink et al., 2020).

However, previous studies of 40 Hz-tACS to improve cognition in AD patients showed that although cognition improved well during the treatment period, the after-effects were insufficient, and the patients’ cognition began to deteriorate gradually after treatment (Zhou et al., 2022). Our study aimed to design a new stimulation method to allow patients to maintain a prolonged after-effect without deterioration of cognition during the non-treatment period. In humans, auditory stimuli of varying gamma frequencies cause an electroencephalogram (EEG) steady-state response (SSR), which cycles with the stimulus frequency and has the greatest amplitude when the stimulus is given at 40 Hz (Galambos et al., 1981). 40 Hz auditory steady state (ASSR) mainly shows neocortical mid-thalamus evoked gamma oscillations in the receptor layer, and ASSR may be a biomarker for detecting cognitive deficits associated with impaired thalamocortical connectivity (Li et al., 2021). Auditory stimulation at 40 Hz has been shown to selectively activate the auditory areas of the pontocerebellum and increase cerebral blood flow to regions of the contralateral auditory cortex, superior temporal gyrus (STG), ipsilateral posterior central gyrus, and inferior temporal gyrus (Pastor et al., 2002).

40 Hz sound stimulation has a positive effect on cognition. 40 Hz auditory stimulation leads to associated protein, glial, and vascular responses in mice model of AD, which have a critical protective effect on neurons. Seven days of one-hour 40 Hz auditory stimulation in AD mice improves memory performance (Martorell et al., 2019). In young people, a 15 min auditory stimulation of 40 Hz can enhance the gamma oscillations in the temporal, frontal and central regions of the brain, and a 20 min auditory stimulation can enhance the working memory performance (Jirakittayakorn and Wongsawat, 2017). Abnormalities in the 40 Hz ASSR can affect cognitive function (e.g., working memory, attention, etc.) (Light et al., 2006; Tada et al., 2016). ASSR at 40 Hz has been proposed as a potential biomarker for schizophrenia (Griskova-Bulanova et al., 2018). Repeated clicks have been shown to produce the strongest and most stable ASSR response, while frequency modulation has the strongest response at about 40 Hz with a significant response (McFadden et al., 2014).

The combination of tACS and sound stimulation may produce synergistic effects, with sound stimulation activating local neurons that overlap with the extensive activation induced by surface electrical stimulation (Conlon et al., 2020; Jones et al., 2020), synergistic stimulation of these two modalities could lead to improved synaptic plasticity with enhanced modulation of local overlapping regions (Meyers et al., 2019; Markewitz et al., 2019). Therefore, tACS combined with sound stimulation is expected to produce long-term after-effects. Our experiment has the following objectives: (1) To study the safety, tolerability, and daily compliance of tACS combined with sound stimulation to treat AD; (2) to determine the effect of tACS combined with sound stimulation on the cognition of AD patients and the maintenance time of after-effects. Furthermore, to explore the effect of the timely and offline effects of the three treatment methods of electrical stimulation combined with sound stimulation, electrical stimulation, or sound stimulation on the cognitive effects of AD patients; (3) to explore the mechanism of the modulatory effect of tACS combined with sound stimulation on the brain network of Alzheimer’s patients by functional magnetic resonance imaging (fMRI).

## Methods and analysis

### Study design

This was a single-center, prospective, randomized, single-blind, and controlled trial. Patients were screened according to strict inclusion and exclusion criteria, and each eligible patient was evaluated at baseline after written informed consent was obtained. Demographic information (e.g., age, gender, race, education, smoking status, alcohol intake, concomitant medications, co-morbidities, surgery, medical history, and fitness level) will also be evaluated at screening.

**Figure 1:**
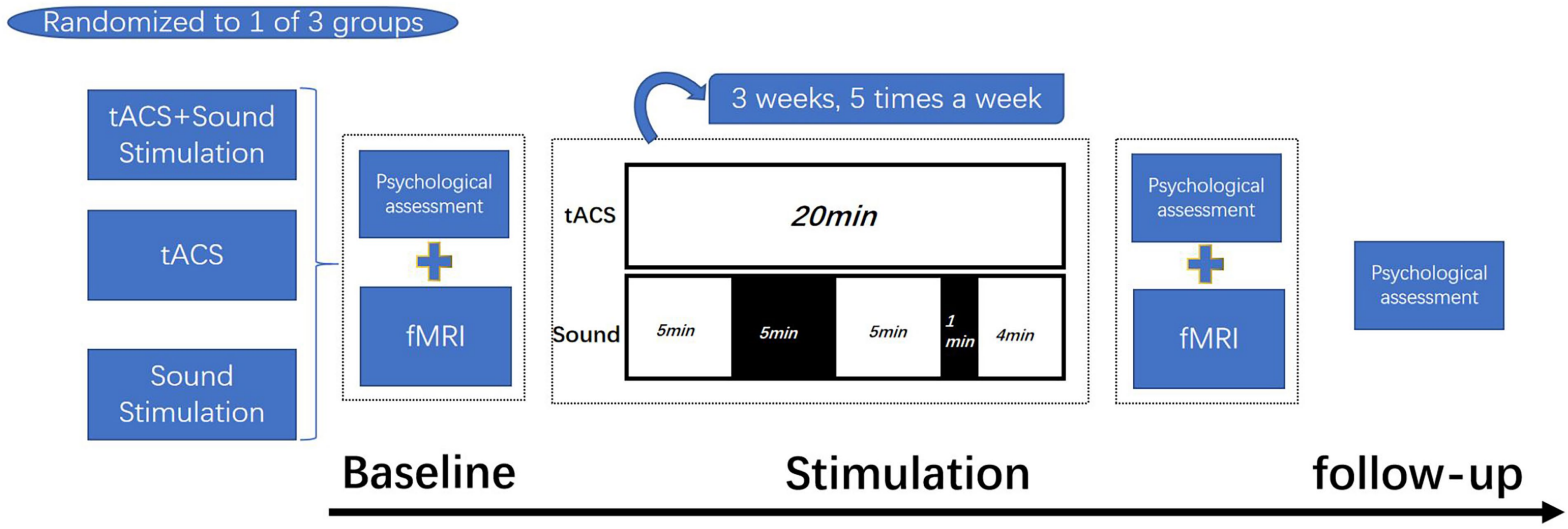
Test process. Patients are randomized into three groups who will receive a three-week (15 sessions) treatment of tACS combined with sound stimulation, tACS, and sound stimulation. tACS: 20 min/day; Sound stimulation: 14 min/day(stimulate for 5 min, rest for 5 min, stimulate for 5 min, rest for 1 min, and stimulate for 4 min). Psychological assessments were made at baseline, after treatment, and at follow-up, and fMRI was applied at baseline and after treatment.

Eighty-seven eligible patients with mild to moderate AD are randomized into three groups who will receive a three-week (15 sessions) treatment of tACS combined with sound stimulation, tACS, and sound stimulation. Efficacy will be assessed at baseline (before the first treatment and on the day of treatment), post-treatment (after the last treatment and on the day of treatment), and the 12-week post-treatment follow-up. Outcomes will include multiple neuropsychological assessments to examine the impact of each treatment on cognitive function before and after treatment and at follow-up, which physicians will perform in the AD Treatment Group of the Department of Neurology at the Chinese People’s Liberation Army (PLA) General Hospital. Brain connectivity and neural activity at rest will be measured by fMRI before and after treatment (Figure 1).

### Methods

#### Sample size

Sample size analysis was based on hypothetical changes in ADAS-Cog scores, and we used to change from baseline to post-treatment or 16-week scores as the primary outcome measure, and for some patients with missing follow-up data, we used their post-treatment data for sample size calculations. In a preliminary clinical trial, we treated two AD patients with tACS combined with sound stimulation and four AD patients with tACS. For comparison between Group A and Group B or between Group A and Group C. We temporarily lack Group C data. Group A (^−^X ± SD) = −6 ± 1; Group B (^−^X ± SD) = 1.75 ± 4.32. We assume that the single-side α error probability is equal to 0.025; Power is 0.9. The sample size needs to be 23 for each group. To allow for a maximum dropout rate of 20%, the sample size is set to 87 participants, 29 in each group.

### Participants

#### Recruitment

We advertise on WeChat public accounts (PLA General Hospital Medical and Health Services and Tianyou Ankang). We mainly included AD patients in various districts of Beijing and a small number of AD patients in other cities in China. Patients interested in this treatment contacted the researchers by phone or WeChat for a first round of screening through verbal questioning. We invite potential participants to undergo final enrollment screening at the Chinese PLA General Hospital.

#### Randomization and blinding

We will generate the block randomization code by computer using the Kangzhi OpenClinica Community Edition automatic grouping module. Only the physician treating the patient will know about the patient grouping, not the other physicians. The researcher performing the cognitive assessment will not be the one performing the treatment, and the patient will be blinded to the stimulus type. Unblinding will not be allowed unless particular clinical circumstances justify it (i.e., necessary for acute medical management of Severity Adverse Events (SAEs)) and only after approval by the principal investigator (PI) or the qualified intermediary.

#### Eligibility criteria

Participants will be evaluated according to the inclusion and exclusion criteria highlighted in Table 1.

**Table 1:** List of inclusion and exclusion criteria.

Inclusion	Exclusion
Age 40–80 years, male or female Meet the diagnosis AD formulated by the National Institute on Aging and the Alzheimer’s Association (NIA-AA) dementia diagnostic criteria Clinical Dementia Rating Scale (CDR) = 1.0 or 2.0 MMSE score (10–26) Able to move freely or with the aid of a walker or crutches Good vision and hearing, able to cooperate with examination and treatment Subjects voluntarily joined and had a guardian sign the informed consent.	The presence of preoperative structural brain abnormalities (such as tumors, cerebral infarction, hydrocephalus, or intracranial hemorrhage) The presence of other neurological disorders such as multiple sclerosis, epilepsy, Parkinson’s disease, etc. Psychiatric system disorders: such as anxiety disorders, affective disorders such as depression, or pharmacogenetic psychiatric disorders Severe medical illness, current use of respiratory medications, cardiovascular medications, anticonvulsants or psychoactive drugs, and clinically significant gastrointestinal, renal, hepatic, respiratory, infectious, endocrine or cardiovascular disease, cancer, alcoholism, or drug addiction Severe hearing (hearing threshold ≤25 dB) and visual impairment Patients with clinical comorbidities with a life expectancy of less than 2 years Patients who have undergone cranial surgery Contraindications to undergoing magnetic resonance imaging or receiving transcranial alternating current stimulation (pacemakers, post-deep brain stimulation (DBS) surgery) Eczema or sensitive skin Familial AD Presence of other types of dementia: vascular dementia, Lewy body dementia, frontotemporal dementia, infectious dementia, etc. Other conditions that, in the opinion of the investigator, may not be suitable for this study.
Withdrawal criteria	
Serious side effects occur, causing safety problems for the patient Patients refuse to continue treatment Lost visits	
Termination criteria	
Continuation of the study may harm the relevant rights and interests of a certain number of subjects	

### Interventions

#### Group A: tACS combined with sound stimulation

tACS instrument (XPNS208-B, Suzhou Hypnos MD Co., Ltd., China). The patients will receive tACS with gamma frequency (40 Hz) and a peak-to-peak amplitude of 1.5 mA 15 times, 20 min sessions across 3 weeks (21 days). The two electrodes (4 × 6 cm^2^) are placed in the dorsolateral prefrontal cortex (DLPFC) and the contralateral supraorbital area, which is located at F3 and F4 based on the 10–20 international Electroencephalogram (EEG). (Two electrodes are placed on the skin surface on the upper edge of the left and right brow arches respectively). The electrodes were placed into a dummy EEG cap corresponding to the international 10–20 EEG system. The electrodes are made of silica gel and sponge. In order to ensure good conductivity of the electrodes, they are soaked in saline before use. Sound Stimulation: Two sponge earbuds are placed in the patient’s ear, and the sound tone is set to 40 Hz (The average interval between pulses was 25 ms), 60 dB, and the patient can hear. Turn on sound stimulation at the same time at the beginning of tACS. The time of sound stimulation is set to stimulate for 5 min, rest for 5 min, stimulate for 5 min, rest for 1 min, and continue stimulation until tACS ends while turning off sound stimulation. The tACS and sound stimulation started and ended simultaneously.

#### Group B: Transcranial alternating current stimulation

The stimulus paradigm of tACS in group B was partially identical to that of tACS in group A. However, only two earplugs were given to the patients, the sound stimulator was not turned on, and the patients did not hear any sound. The patients did not know whether the sound stimulator was turned on.

#### Group C: Sound stimulation

The sound stimulation paradigm in this group of patients was the same as in group A. Total duration was 20 min. The appearance of the sham tACS stimulator was identical to that of the tACS stimulator. However, no current flowed through the electrodes when the device was activated.

## Outcome measurements

### Main outcome

ADAS-Cog score: ADAS-Cog will evaluate the changes in the global cognitive function pre-and post-intervention and follow-up. ADAS-cog scale ranges from 0 to 70, and a higher value represents a worse outcome. The ADAS-Cog examination contains the primary cognitive function measures specified by the dementia diagnostic criteria and is widely used in drug clinical trials to evaluate cognitive change. A 4-point improvement in the score (equivalent to a 6-month average natural decline score) is generally used as a criterion for efficacy. However, the ADAS-Cog is not suitable for the assessment of very mild and very severe dementia (Rosen et al., 1984).

### Secondary outcome

#### Cognitive assessments

MMSE score: Mini-mental State Examination (MMSE) will evaluate the changes in the general cognitive function pre-and post-intervention and follow-up. MMSE ranges from 0 to 30, and a higher value represents a better outcome. It consists of five dimensions, including orientation (10 points), registration (3 points), attention and calculation (5 points), recall (3 points), and language and praxis (9 points). The MMSE is a 30-point scale, with a score of 27–30 indicating normal cognitive function and a score of <27 suggesting cognitive disorder. The severity of dementia is graded as per MMSE score: mild (≥21 points), moderate (10–20 points), and severe (≤9 points) (Cockrell and Folstein, 1988).

MoCA score: Montreal Cognitive Assessment (MoCA) will evaluate the changes in the general cognitive function pre-and post-intervention and follow-up. MoCA ranges from 0 to 30, and a higher value represents a better outcome. The MoCA includes executive functioning, language, orientation, computation, abstract thinking, memory, visual perception, attention, and concentration. The scale is strongly influenced by education level. The MoCA test takes about 15 min and has a total score of 30 (Nasreddine et al., 2005).

CDR score: Clinical Dementia Rating (CDR) will be used to evaluate the level of dementia in AD patients. The CDR is used to characterize six domains (memory, orientation, judgment and problem solving, community affairs, home and hobbies, and personal care) of the cognitive and functional performance of the aged (AD patients in particular). The information is obtained through semi-structured interviews with the patient and a reliable informant or collateral source (e.g., a family member). Patients are rated for dementia severity: 0 = normal, 0.5 = questionable dementia, 1 = mild dementia, 2 = moderate dementia, and 3 = severe dementia (Morris, 1993).

AVLT score: Auditory verbal learning test (AVLT) will be used to evaluate the changes in the memory function pre-and post-intervention and follow-up. AVLT ranges from 0 to 36, and higher values represent better outcomes. AVLT is the most commonly used scale to check episodic memory. For a total of 12 words, all the words read guidance subjects immediately after one times memories, memories and record the number of words, the exact repeat three times, as the “immediate recall, “told the participants to remember these words after learning, three times in 5 min and 20 min after guidance subjects recalled, and record the correct number, Short delayed recall and long-delayed recall, respectively. Finally, subjects were instructed to recall and recognize words (Geffen et al., 1994).

BNT-30 score: Boston Naming Test (BNT-30) will be used to assess the changes in the language function pre-and post-intervention and follow-up. It ranges from 0 to 30, and a higher value represents a better outcome. The BNT-30 is widely used to assess language function in various cognitive disorders. BNT can screen for AD, and test scores are not affected by age or education.

#### Psychiatric symptoms assessment

NPI score: The Neuropsychiatric Inventory (NPI) will be used to measure the changes in the neuropsychiatric symptoms pre-and post-intervention and follow-up. It ranges from 0 to 144, and a higher value represents a worse outcome. The higher the NPI score, the worse the patient’s mental state. The NPI scale, which measures ten common behavioral disorders in dementia patients, is often used to evaluate the efficacy of drugs on psychiatric symptoms and to identify the causes of dementia. The caregiver answers. It takes 7–10 min (Cummings et al., 1994).

HAMA score: The Hamilton Anxiety Scale (HAMA) will measure the changes in the anxiety symptoms pre-and post-intervention and follow-up. If the total score is ≥29, severe anxiety is possible; if the score is ≥21, significant anxiety is present; if the score is ≥14, anxiety is present; if the score is more than 7, anxiety is probably present; if the score is less than 7, no anxiety symptoms are present.

BDI score: Beck Depression Inventory (BDI) will be used to measure the changes in the depression situations pre-and post-intervention and follow-up. Higher scores indicate higher levels of depression. BDI total score ≤4, no depression or very mild; 5< total score <13, mild; 14< total score <20, moderate; total score >21, severe.

#### Quality of life assessment

ADL score: Activities of Daily Living (ADL) scale will be used to assess the change in life quality pre-and post-intervention and follow-up. It ranges from 20 to 80. The “20” represents average life ability, and the higher score presents the worse life ability (Upton, 2013).

### Assessment of brain network connectivity and mechanisms

Participants will receive brain magnetic resonance imaging (MRI) at baseline at the end of the intervention. MRI can effectively reflect the structural changes of gray matter and white matter in patients with AD, the parameters of the brain structure network (clustering coefficient, characteristic path length and node coefficient, etc.,) and the change of cerebral cortex thickness. fMRI can show longitudinal connectivity changes in the anterior temporal network, and this brain network connection is closely related to cognition (Dautricourt et al., 2021).

T1-weighted images of the whole brain will be obtained using a sagittal three-dimensional (3D) magnetization prepared rapid gradient echo (MPRAGE) sequence: repetition time (TR) = 2,530 ms; echotime (TE) = 2.02 ms; slice thickness = 1 mm; flip angle = 7°; field of view (FOV) = 300 × 300 mm^2^; slice number = 192; Inversion time TI = 1,100; Voxel size = 1.0 × 1.0 × 1.0 mm.

Resting-state fMRI will be conducted using a multiband echo-planar imaging (EPI) sequence: TR = 2020 ms; TE = 30 ms; slice number = 33; slice thickness = 3.5 mm; gap = 1 mm; flip angle = 90°; FOV = 300 × 300 mm^2^; Voxel size = 3.5 × 3.5 × 3.5 mm.

### Adverse events

Side effects will be evaluated after each treatment. Patients will be asked whether they have the following conditions and whether they can tolerate them. Adverse reactions include headache, itching, dizziness, burning sensation, skin redness, neck pain, tinnitus, lethargy, inattention, acute emotional changes, flashing lights, and other symptoms. If the patient cannot tolerate the adverse reactions, the treatment of the patient will be terminated (Table 2).

**Table 2:** Outcome assessment schedule.

	At baseline	End of intervention	3 months follow-up
**Primary outcome measure**			
ADAS-Cog	×	×	×
**Secondary outcome measures**			
MoCA	×	×	×
MMSE	×	×	×
CDR	×	×	×
AVLT	×	×	×
NPI	×	×	×
HAMA	×	×	×
BDI	×	×	×
BNT-30	×	×	×
ADL	×	×	×
MRI	×	×	
Side-effects of tACS	After each treatment		

## Data management and analysis

### Data management

After recruitment, researchers will replace the names of participants with a 4-letter code to protect their privacy. Paper-based data consist of the original assessment form and Case Report Form (CRF) at baseline, post-treatment, and 12 weeks post-treatment. They are stored at the Department of Neurosurgery, PLA General Hospital. All electronic data will be deposited in the Kangzhi OpenClinica Community Edition database. Original documents, CRFs, and other records related to this study will be retained for 10 years.

### Data analysis

All calculations will be performed using IBM SPSS Statistics 25.0. Descriptive statistics for demographic and baseline characteristics will provide means and standard deviations, and all data will be analyzed according to the intention-to-process principle. To explore the demographic characteristics of participants, independent sample tests for continuous data and chi-square tests for dichotomous variables (using Fisher’s exact test, if needed) will be used.

The effect of tACS on neuropsychological scores and MRI data will be examined using linear mixed-effects models nested within individuals. Time will be specified as a repeated variable. Group, time, and group by time will be included as fixed effects. We will analyze changes in outcomes from the baseline examination to the end of the intervention and from the baseline examination to the 12-week post-treatment follow-up. For adverse events, a chi-square test or Fisher’s exact test will be used to compare the frequencies between the groups. Correlation analyses between significant neuroplasticity changes and neuropsychological scores will be performed to explore the neural mechanisms underlying changes in cognitive function. For all analyses, the significance level will be set at 0.05.

### Data monitoring

This study will be supervised by the Ethics Committee of the PLA General Hospital, whose members do not have any conflict of interest with this study. The principal investigator will have access to all results and make the final decision to terminate the study. They will review all Adverse Events (AEs), SAEs, protocol deviations, study progress, and audit study procedures if required. Protocol revisions will be reported to this committee. All information related to AEs, SAEs, protocol revisions, and protocol deviations will be reported to the appropriate research ethics committee. In addition, they will grant project members the right to disseminate trial results through published papers.

## Discussion

The combined treatment strategy, tACS combined with sound stimulation, may have greater and longer-lasting efficacy in improving cognition in patients with AD. This was also confirmed in our previous trial: a patient with moderate AD not only showed improvement in cognitive scale scores after 15 sessions of tACS combined with sound stimulation but also continued to improve cognitive function at the 4-month follow-up. Scores on ADAS-Cog decreased by 7 points at follow-up compared to baseline levels. Scores on MoCA and MMSE improved (Liu Y. et al., 2022). However, the available clinical studies on tACS in the treatment of AD patients and the results of our pilot test show that tACS has a timely effect on improving cognition in AD patients. In addition, there are few clinical trials to study the after-effect, and the only studies on the after-effect have not achieved good results (Zhou et al., 2022). For example, in two AD patients treated with 40 Hz-tACS for 14 weeks, there was a potential improvement in patient cognition after treatment (Bréchet et al., 2021). 60 min of single 40 Hz tACS in areas located in the medial parietal cortex and precuneus improved memory function in patients with mild cognitive impairment (Benussi et al., 2021). Patients with mild cognitive impairment and mild to moderate dementia treated with twice-daily 40 Hz tACS for 4 weeks were found to have a trend towards improved memory after treatment and at the one-month follow-up (Kehler et al., 2020). Six weeks of 40 Hz tACS (2 mA, 20 min) and patients’ cognitive scale scores improved after treatment. However, a trend towards deterioration in both scale scores compared to post-treatment was found at the 12-week follow-up (Zhou et al., 2022). AD patients were treated with tACS (40 Hz, 4 mA) for 2 or 4 weeks for 1 h each time. The stimulation site was located in the bilateral temporal lobe. The results showed that there was no significant change in overall cognition after tACS (Sprugnoli et al., 2021).

To our knowledge, the use of electrical stimulation combined with sound stimulation to improve cognition in patients with mild to moderate AD has been proposed for the first time. This treatment approach is expected to better improve cognitive function in AD patients, especially in some cognitive domains of particular interest, including attention, orientation, and memory, and has long-term offline effects not available with 40 Hz-tACS alone. We relied on multiple cognitive-related scales to measure multiple aspects of cognition in patients. According to our pilot test results, after tACS in the DLPFC region, the patients’ word recognition and delayed recall have improved significantly. The reason for this effect may not only use 40 Hz stimulation frequency but also may have a great correlation with the stimulation site we choose. The DLPFC region may have the ability to adapt to neural interventions derived from the body. Because of its extensive connections with subcortical areas (Frith and Dolan, 1996). The DLPFC region is involved in the regulation of various cognitive functions such as memory and attention. And plays an important role in memory initiation, information consolidation, and processing (Bahmani et al., 2019). And the DLPFC region has an impact on psychoneurological emotion, so we evaluated the patients with three scales of NPI, HAMA, and BDI before and after treatment and during follow-up.

Another feature of this trial is that in addition to evaluating patients on several neuropsychological scales, we make MRI comparisons of patients before and after treatment in this trial. We used structural MRI to assess structural changes in the patient’s brain before and after treatment, and it is well known that AD patients have varying degrees of gray matter degeneration and white matter lesions in the brain (Brun and Englund, 1986). And fMRI was used to analyze the changes in brain network connectivity of patients before and after treatment. fMRI, a resting state, allows for studying the functional activity of two or more functional brain regions (Al-Sharoa et al., 2019). Several papers have reported abnormalities in default functional network connectivity in patients with AD (Allen et al., 2007; Subramanian et al., 2020; Zhao et al., 2020; Sedghizadeh et al., 2022), and the strength of functional connectivity has a more significant correlation with cognitive scale scores (Grieder et al., 2018).

Since the drugs developed today for the treatment of AD do not improve the cognition well of patients and considering the physical tolerance of AD patients to surgery, non-invasive brain stimulation treatments are the future trend in the treatment of AD (Eissa et al., 2022; Haddad et al., 2022). tACS may activate excitable peripheral components between the scalp electrodes, including the trigeminal branches, the greater occipital nerve, the retina, and the vestibular organs (Schutter and Hortensius, 2010). Some adverse reactions can occur, such as phosphene, Dizziness, skin sensation, pressure perception, etc., However, all reported adverse events of tACS so far have been transient rather than persistent (Turi et al., 2013; Raco et al., 2014; Matsumoto and Ugawa, 2016). Cognition in AD patients deteriorates gradually without treatment, and today’s non-invasive brain stimulation methods do not have a long-term offline effect on AD patients’ cognition, although they can improve it in the short term. In contrast, our design solution is to administer tACS and sound stimulation simultaneously to patients. When electricity is combined with sound to stimulate the brain, the brain becomes more active and also activates a large number of neurons in the cerebral cortex. Only two studies have shown that sound stimulation combined with electrical stimulation can be a good treatment for tinnitus (Conlon et al., 2020) and chronic pain (Gloeckner et al., 2022). And it has long-term after-effects. To our knowledge, there is no effective treatment for these two disorders so far, and the use of electrical stimulation combined with sound stimulation is a breakthrough. Therefore, tACS combined with sound stimulation in the treatment of AD is also expected to produce long-term after-effects. To verify whether this offline effect of dual stimulation is better than that of a single stimulation modality, we added a tACS alone group and a sound stimulation alone group for efficacy comparison.

There are some limitations in our trial protocol design. First: the tACS group does not produce any sound from the sound stimulation apparatus even though the patients are given the same sponge earplugs, this may result in the patients knowing that they are not receiving tACS combined with sound stimulation treatment and not achieving complete patient blindness. However, our protocol was designed to explore the long-term effects of tACS combined with sound stimulation on the cognition of AD patients, so there were no strict requirements for participants to be completely unaware of this issue. Second: Since the electrical stimulation instrument and the sound stimulation instrument are two machines, there may be a slight time difference when two doctors turn on the two machines at the same time, but whether this time difference will affect the synchronization of neurons, we do not know yet. Maybe in the future, when this treatment method becomes more and more mature, we can also manufacture this all-in-one machine for synchronous stimulation of sound and electrical stimulation. Third: Although the results in our pilot test showed that the method of sound stimulation combined with electrical stimulation would produce better cognitive improvement effects, this result appeared due to “the effect of sound stimulation + the effect of electrical stimulation” Or “the effect of the interaction of sound stimulation and electrical stimulation” is still unclear for the time being. Perhaps doing an EEG on the patient alongside treatment would explore whether the two stimuli interact. However, the purpose of this trial is to explore the clinical efficacy of tACS combined with sound stimulation as a new therapeutic approach to affect the cognition of AD patients. In the future, when this treatment method becomes more and more mature, we will better solve these problems.

In conclusion, in the face of today’s trend of increasing morbidity and mortality in AD patients, it is of great interest to develop treatments that can slow down the progression of AD disease, improve the condition of AD patients and have offline effects. This “multimodal” combined stimulation approach may be applied to the treatment of more neurological disorders in the future. This study provides a new, structured framework for moving forward.

## Data availability statement

The raw data supporting the conclusions of this article will be made available by the authors, without undue reservation.

## Ethics statement

Ethics approval for this trial (approval no. S2019-250-02) was obtained from the Ethics Committee of Chinese PLA General Hospital. These research ethics boards will approve all subsequent amendments to the protocol. If participants did not have the cognitive capacity to consent, their appointed powers of attorney provide written informed consent before their participation in the trial. Participants will be informed of any changes or new information that may affect their safety and willingness to continue participating in the study.

## Author contributions

YL, SL, CT, KT, DL, MC, ZM, and XX contributed to the study conception and design. AD patients were enrolled and treated by YL and DL. Diagnosis of AD patients is performed by ZM. Data collection and analysis were performed by KT and MC. The first draft of the manuscript was written by YL and SL. The experiment was designed by YL, XX, and ZM. The scale was evaluated by YL. English translation by CT. All authors contributed to the article and approved the submitted version.

## Funding

This study was supported by grants from the China Brain Project (2021ZD0200407), the National Natural Science Foundation of China (no. 81871087), the Innovative Technique Project of Chinese PLA General Hospital (XJS-202103), and the National Clinical Research Center for Geriatric Diseases (no. NCRCG-PLAGH-2018006).

## Conflict of interest

DL, KT, and MC were employed by Guangzhou Kangzhi Digital Technology Co., Ltd.

The remaining authors declare that the research was conducted in the absence of any commercial or financial relationships that could be construed as a potential conflict of interest.

## Publisher’s note

All claims expressed in this article are solely those of the authors and do not necessarily represent those of their affiliated organizations, or those of the publisher, the editors and the reviewers. Any product that may be evaluated in this article, or claim that may be made by its manufacturer, is not guaranteed or endorsed by the publisher.
